# Supplementary material for: Vaccination with CD47 deficient tumor cells elicits an antitumor immune response in mice
Source: Nat Commun. 2020 Jan 29;11:581. doi: 10.1038/s41467-019-14102-4 (PMC6989506; doi:10.1038/s41467-019-14102-4)
Supplement: Supplementary file 1 — Supplementary Information [file 41467_2019_14102_MOESM1_ESM.pdf]

SUPPLEMENTARY INFORMATION

Vaccination with CD47 deficient tumor cells elicits an antitumor immune response in mice

Yang Li *et al*.

Contents: Supplementary Figures 1-9

Supplementary Figure 1

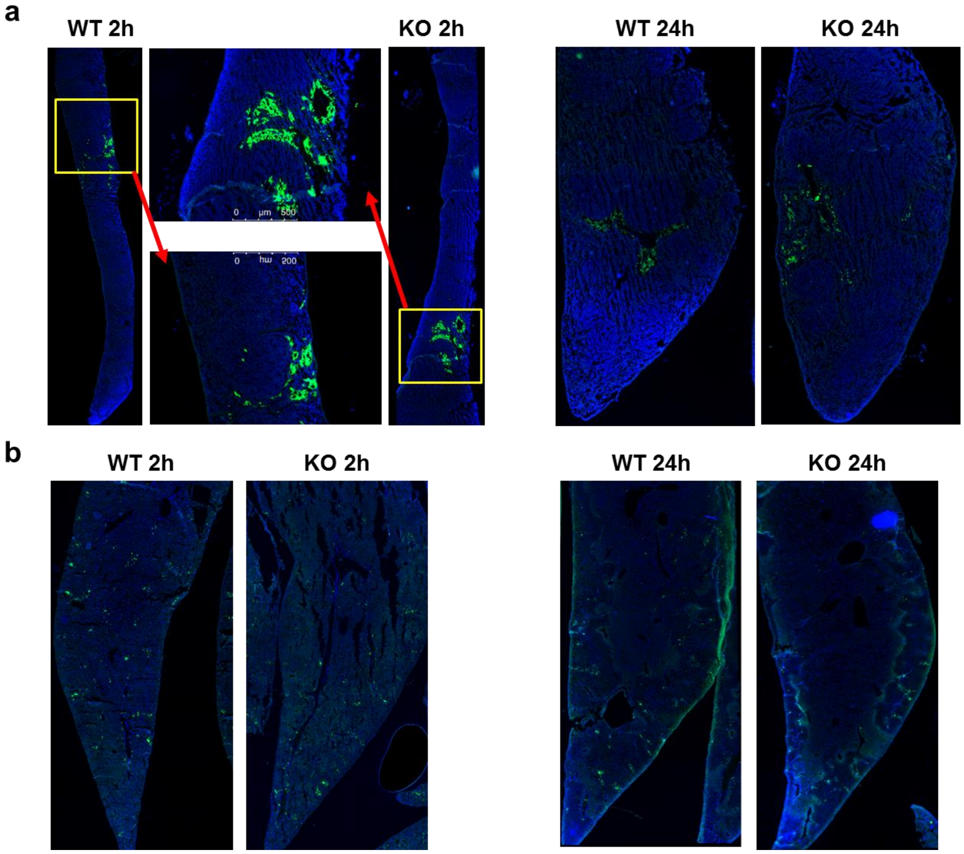

Supplementary Figure 1. Tissue distribution of intrasplenically injected tumor cells. WT and CD47KO B16F0 cells were labeled with CFSE and irradiated (70Gy), then injected into the spleen of B6 mice (2.5×10^6 per mouse). Spleen and liver tissues were harvested 2h and 24h after tumor cell injection, fixed and sectioned (5μm) along vertical or horizontal axis, and mounted with mounting medium with DAPI (Vector Laboratories). Images were obtained using Leica DMI 6000B wide field microscope. Each group contained 3 mice, and images of spleen (**a**) and liver (**b**) from representative mice are presented.

Supplementary Figure 2

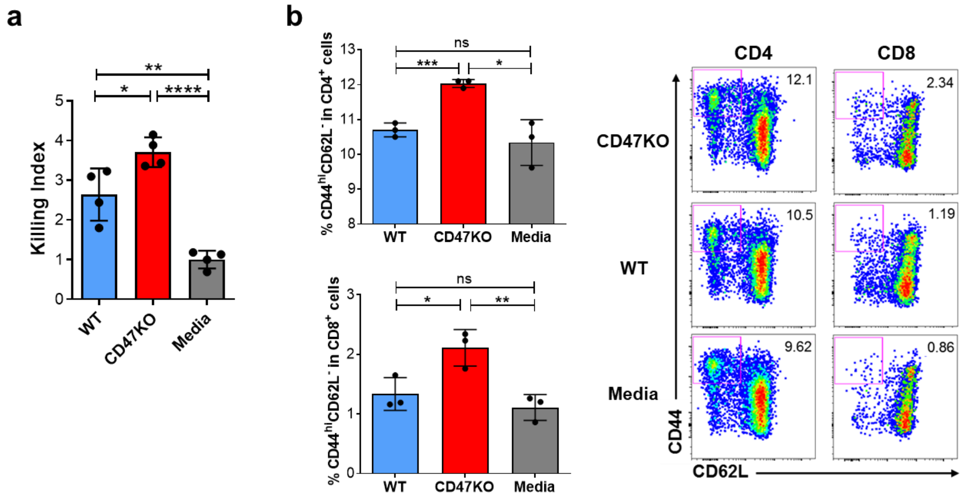

Supplementary Figure 2. CD47KO tumor cell vaccination results in enhanced killing of tumor cells and increased frequency of effector/memory T cells. Spleen cells were harvested from mice 7 days after vaccination with irradiated WT or CD47KO B16F0 cells or media controls, and examined for killing of B16F0 cells by *in vitro* killing assay and frequency of $CD44^{hi}CD62L^{low/-}$ cells by flow cytometry. (**a**) Killing activity shown as percentages of PI^{+} cells normalized to the media control (mean±SDs; n=4 per group). (**b**) Percentages (Left; mean±SDs; n=3 per group) and staining profiles (Right) of $CD44^{hi}CD62L^{low/-}$ cells in the $CD4^{+}$ and $CD8^{+}$ T cell populations. *, $p < 0.05$; **, $p < 0.01$; ***, $p < 0.005$; ns, not significant (unpaired t-test).

Supplementary Figure 3

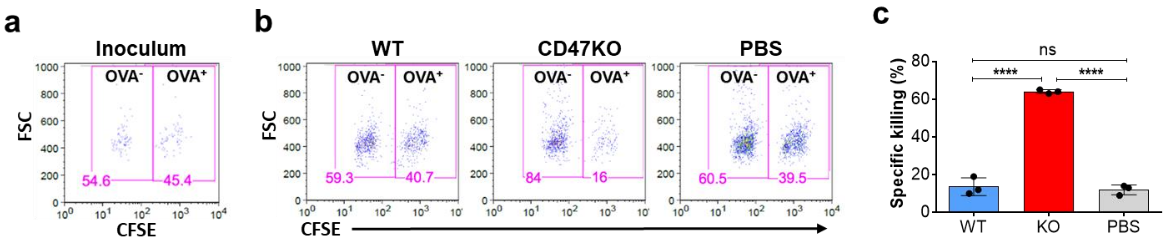

Supplementary Figure 3. Specific killing of OVA+ cells measured by in vivo killing assay. C57BL/6 mice received intrasplenic injection of splenocytes from WT or CD47KO OVA-Tg C57BL/6 mice or PBS, followed 10 days later by injection (i.v.) of a mixture (1:1) of OVA$^+$ and OVA$^-$ B6 mouse splenocytes that were labelled with different CFSE intensity. The ratio of OVA$^+$ (CFSE^{hi}) to OVA$^-$ (CSFE^{lo}) cells in the recipient spleen was measured by flow cytometry 16 hours after cell injection, and specific killing (%) of OVA$^+$ cells was calculated as [% CFSE^{hi} in the inoculum - % CFSE^{hi} in the spleen)/(% CFSE^{hi} in the inoculum)] × 100. (**a**) Flow cytometry profiles of mixed CFSE-labelled OVA$^+$ and OVA$^-$ cell inoculum. (**b**) Flow cytometry profiles of splenocytes from representative mice receiving WT or CD47KO OVA-Tg mouse splenocytes or PBS. (**c**) Specific killing (mean±SEM; n=3 per group) of OVA$^+$ cells in the indicated groups. ****, p<0.0001; ns, not significant (unpaired t-test).

Supplementary Figure 4

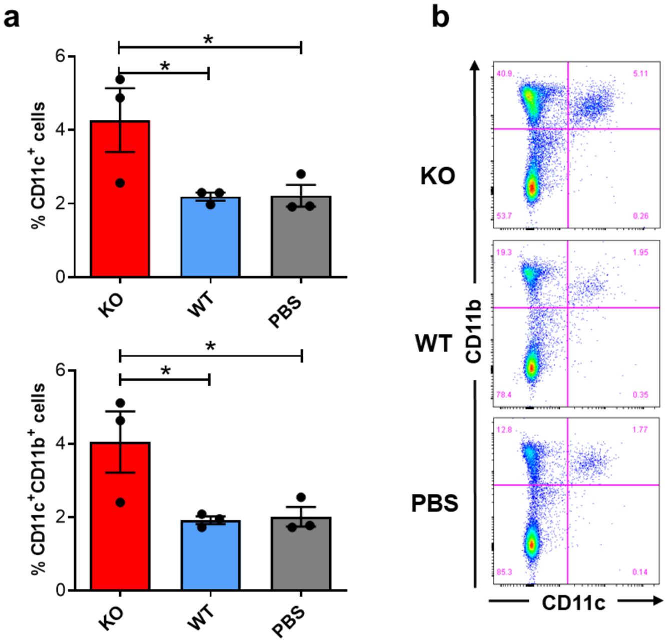

Supplementary Figure 4. CD11c$^+$ cell expansion in mice vaccinated with OVA$^+$ CD47KO splenocytes. Flow cytometry was performed on PBMCs 3 days after intrasplenic injection of PBS, WT or CD47KO OVA$^+$ splenocytes. (**a**) Percentages (mean±SEM; n=3 per group) of CD11c$^+$ (top) and CD11c$^+$CD11b$^+$ (bottom) cells (*, $p<0.05$; unpaired t-test). (**b**) Representative flow cytometry profiles.

Supplementary Figure 5

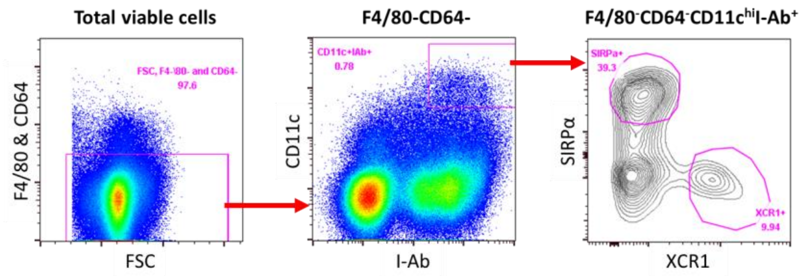

Supplementary Figure 5. Gating strategy for sorting of DCs by flow cytometry. Spleen cells were stained with antibodies specific for mouse F4/80, CD64, CD11c, I-Ab, XCR1, and SIRPα, and then SIRPα^{+}XCR1^{-} and SIRPα^{-}XCR1^{+} DCs were sorted using a BD Influx Cell Sorter. Shown are representative profiles showing expression of macrophage markers F4/80 and CD64 on total viable cells (left), expression of CD11c and I-Ab on F4/80^{-}CD64^{-} cells (middle), and expression of SIRPα and XCR1 on F4/80^{-}CD64^{-}CD11c^{+}I-Ab^{+} DCs.

Supplementary Figure 6

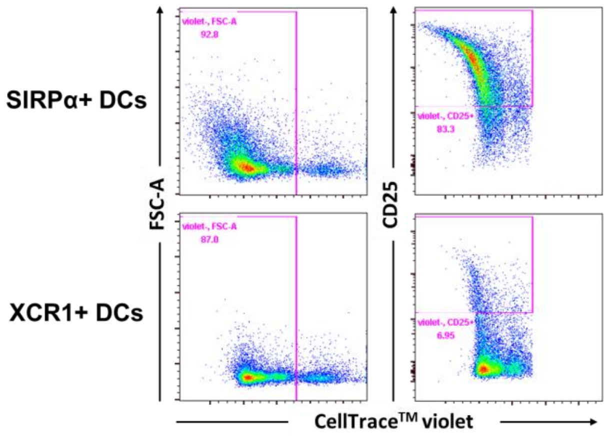

Supplementary Figure 6. SIRPα$^+$ DCs are superior to SIRPα$^-$ DCs in stimulating antigen-specific T cell responses. Flow cytometry-sorted splenic SIRPα$^+$ (XCR1$^-$CD11c$^+$I-Ab$^+$F4/80$^-$CD64$^-$) or XCR1$^+$ (SIRPα$^-$CD11c$^+$I-Ab$^+$F4/80$^-$CD64$^-$) DCs (Supplementary Figure 5) were pulsed with 100 pM OVA peptides (aa257-264), co-cultured with sorted OT-I mouse splenic CD3$^+$CD8$^+$ T cells (pre-labeled with Celltrace^{Tm} violet) for 3 days, then analyzed by follow cytometry for Violet fluorescence dilution in T cells (i.e., proliferation) and CD25 expression on Violet$^{-/low}$ T cells (n=2 per group). Shown are representative flow cytometry profiles revealing Violet fluorescence dilution in T cells (*left*) and CD25 expression on gated Violet$^{-/low}$ T cells (*right*) that were stimulated by SIRPα$^+$ (*top*) or XCR1$^+$ (*bottom*) DCs.

Supplementary Figure 7

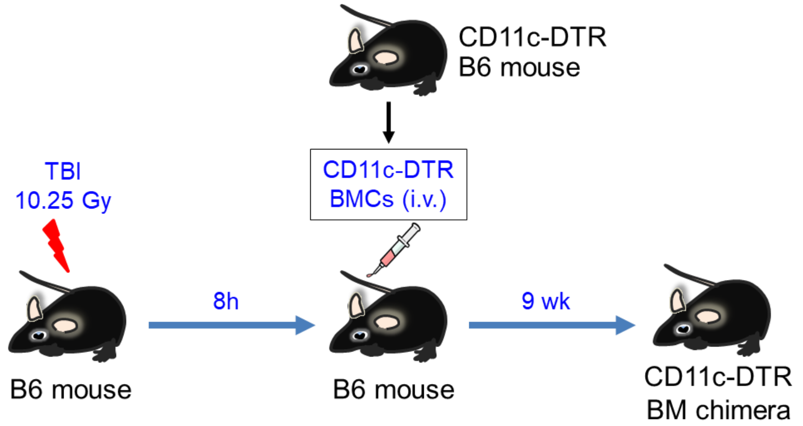

Supplementary Figure 7. Schematic representation of CD11c-DTR bone marrow (BM) chimeras. C57BL/6 (B6) mice were conditioned by total body irradiation (TBI, 10.25 Gy), followed 8h later by intravenous injection (i.v.) of 1×10^7 BM cells from CD11c-DTR B6 mice. The established CD11c-DTR BM chimeras were used for tumor challenge and vaccination 9 weeks after BM cell transplantation (see Figure 5).

Supplementary Figure 8

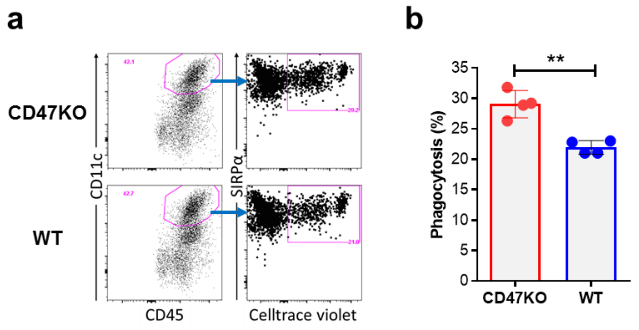

Supplementary Figure 8. More effective endocytosis of CD47KO than WT melanoma cells by SIRPα$^+$ DCs. B6 mouse bone marrow (BM) cells (2×10^6 in 10mL) were cultured in 10% FBS RPMI media containing 20ng/ml mouse GM-CSF for 10 days (with semi medium change at days 3 and 6), then non-adherent and loosely adherent cells were harvested and used as DCs. For measuring endocytosis, CD47KO and WT B16F0 target cells were labeled with 2uM Celltrace^{TM} Violet for 30 mins, and then 1×10^5 labeled tumor cells were co-cultured with 5×10^4 BM-derived DCs for 2h. At the end of culture, cells were stained with fluorescence-conjugated antibodies for mouse CD45, CD11c and SIRPα, and endocytosis was determined by measuring the percentage of Violet$^+$ DCs within the CD45$^+$CD11c^{hi}SIRPα$^+$ cell gate. (**a**) Flow cytometry profiles showing staining of the cultured cells with anti-CD45 and anti-CD11c (*left*) and expression of SIRPα and Violet in gated CD11c^{hi}CD45$^+$ DCs (*right*; almost all CD11c^{hi}CD45$^+$ cells were SIRPα$^+$). (**b**) Levels of endocytosis (i.e., percentages of SIRPα$^+$Violet$^+$ cells among gated CD11c^{hi}CD45$^+$ cells). Results are presented as mean±SD (n=4 per group; **, p = 0.01 by unpaired t-test).

Supplementary Figure 9

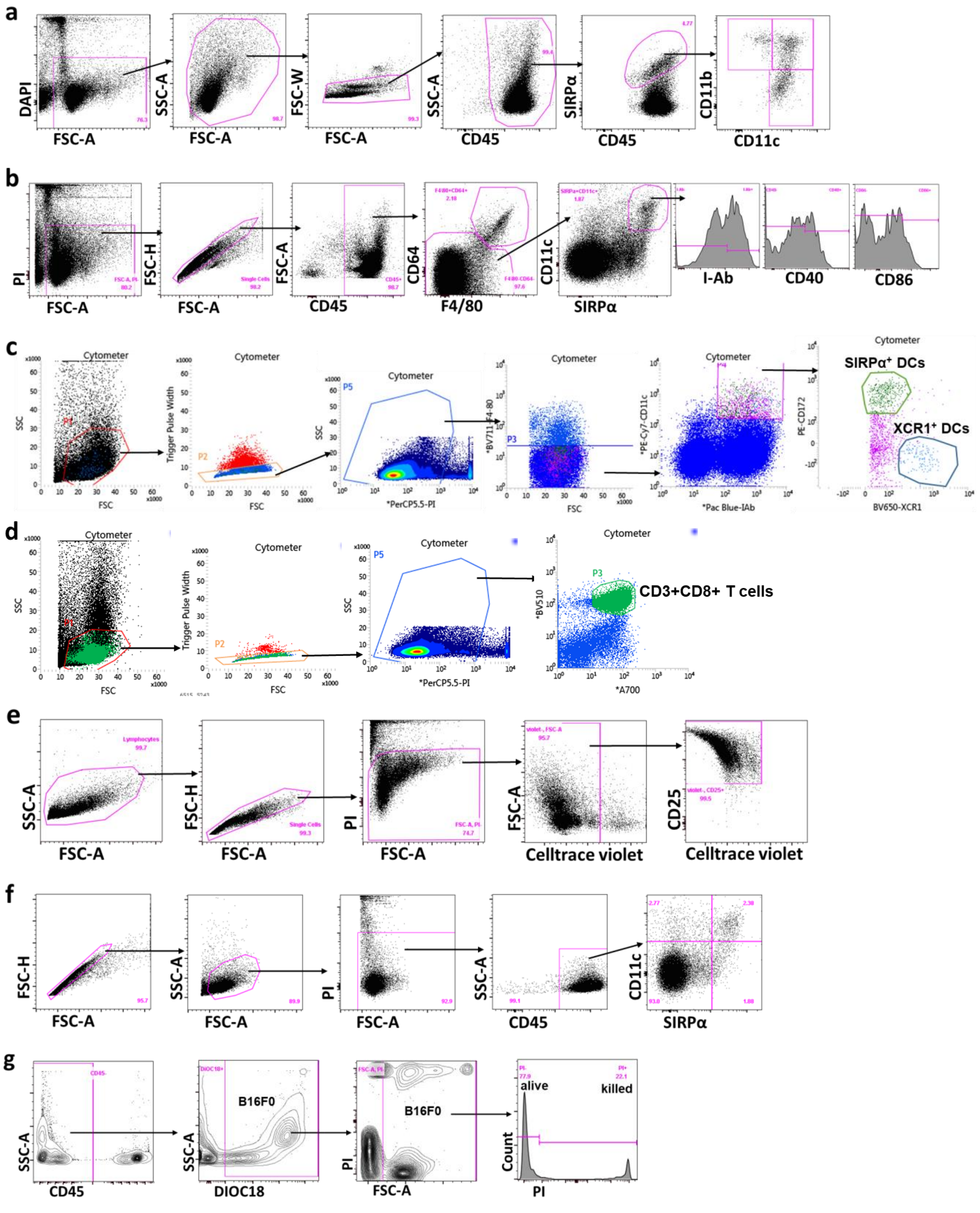

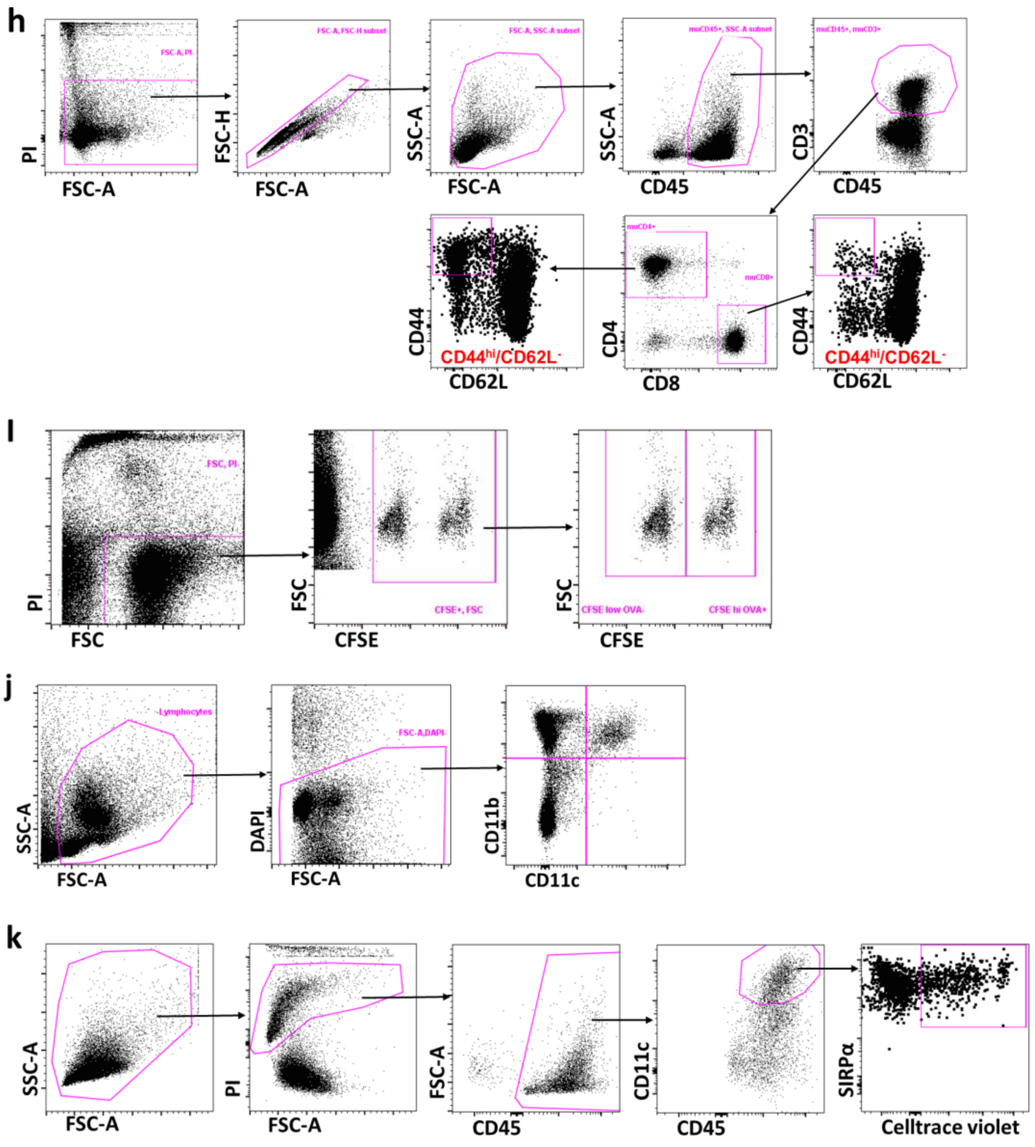

Supplementary Figure 9. FACS sequential gating/sorting strategies. Shown are FACS gating strategies for analyzing SIRPα+ DCs in Figure 4a (**a**), Figure 5a (**b**), and Supplementary Figure 5 (**c**); sorting CD3+CD8+ cells from OT-I mice that were used in the experiments in Figure 6 (**d**); analyzing CD3+CD8+ OT-I T cell activation and proliferation in Figure 6 (**e**); measuring CD11c+ DC depletion in Figure 7b (**f**); measuring killing of B16F0 cells in Supplementary Figure 2a (**g**); phenotypic analysis of activated T cells in Supplementary Figure 2b (**h**); measuring specific killing of OVA+ cells in Supplementary Figure 3 (**i**); detecting CD11c+ DC expansion in Supplementary Figure 4 (**j**); measuring endocytosis of tumor cells by BM-derived SIRPα+ DCs in Supplementary Figure 8 (**k**).
