# Supplementary material for: Vaccination with CD47 deficient tumor cells elicits an antitumor immune response in mice
Source: Nat Commun. 2020 Jan 29;11:581. doi: 10.1038/s41467-019-14102-4 (PMC6989506; doi:10.1038/s41467-019-14102-4)
Supplement: Supplementary file 3 — Reporting Summary [file 41467_2019_14102_MOESM3_ESM.pdf]

nature research

Corresponding author(s): Yong-Guang Yang

Reporting Summary

Nature Research wishes to improve the reproducibility of the work that we publish. This form provides structure for consistency and transparency in reporting. For further information on Nature Research policies, see Authors & Referees and the Editorial Policy Checklist.

Statistical parameters

When statistical analyses are reported, confirm that the following items are present in the relevant location (e.g. figure legend, table legend, main text, or Methods section).

n/a	Confirmed	
☐	☒	The exact sample size (*n*) for each experimental group/condition, given as a discrete number and unit of measurement
☐	☒	An indication of whether measurements were taken from distinct samples or whether the same sample was measured repeatedly
☐	☒	The statistical test(s) used AND whether they are one- or two-sided<br>*Only common tests should be described solely by name; describe more complex techniques in the Methods section.*
☒	☐	A description of all covariates tested
☐	☒	A description of any assumptions or corrections, such as tests of normality and adjustment for multiple comparisons
☐	☒	A full description of the statistics including central tendency (e.g. means) or other basic estimates (e.g. regression coefficient) AND variation (e.g. standard deviation) or associated estimates of uncertainty (e.g. confidence intervals)
☐	☒	For null hypothesis testing, the test statistic (e.g. *F*, *t*, *r*) with confidence intervals, effect sizes, degrees of freedom and *P* value noted<br>*Give P values as exact values whenever suitable.*
☒	☐	For Bayesian analysis, information on the choice of priors and Markov chain Monte Carlo settings
☒	☐	For hierarchical and complex designs, identification of the appropriate level for tests and full reporting of outcomes
☒	☐	Estimates of effect sizes (e.g. Cohen's *d*, Pearson's *r*), indicating how they were calculated
☐	☒	Clearly defined error bars<br>*State explicitly what error bars represent (e.g. SD, SE, CI)*

Our web collection on statistics for biologists may be useful.

Software and code

Policy information about availability of computer code

Data collection	Flow cytometry data was collected by FACSDiva (BD Fortessa). ELISPOT data was collected by Bioreader 5000 software. Fluorescent images were obtained using Leica Application Suite (LAS) software.
Data analysis	Flow cytometry data was analyzed using Flowjo v10 (Tree Star). Graphs and statistical analysis were generated by Prism 7 (Graph pad).

For manuscripts utilizing custom algorithms or software that are central to the research but not yet described in published literature, software must be made available to editors/reviewers upon request. We strongly encourage code deposition in a community repository (e.g. GitHub). See the Nature Research guidelines for submitting code & software for further information.

Data

Policy information about availability of data

All manuscripts must include a data availability statement. This statement should provide the following information, where applicable:

- Accession codes, unique identifiers, or web links for publicly available datasets
- A list of figures that have associated raw data
- A description of any restrictions on data availability

The data that support the findings of this study are available from the corresponding author upon request.

Field-specific reporting

Please select the best fit for your research. If you are not sure, read the appropriate sections before making your selection.

- [x] Life sciences
- [ ] Behavioural & social sciences
- [ ] Ecological, evolutionary & environmental sciences

For a reference copy of the document with all sections, see nature.com/authors/policies/ReportingSummary-flat.pdf

Life sciences study design

All studies must disclose on these points even when the disclosure is negative.

Sample size	For experiments in this study, a reasonable sample size was determined to ensure adequate reproducibility and statistically significant difference, which was based on our and others previous studies.
Data exclusions	There were no excluded data from the analysis in this study.
Replication	All mouse in vivo experiments were performed in multiple times, data was combined by multiple replicates with reproducible results.
Randomization	Age and sex matched animals were randomly assigned to different experimental groups.
Blinding	For mouse in vivo experiments, researchers were blinded to perform the tumor size measurement. The mice were randomized to assign groups and collect data for animal experiments. For those flow cytometry data were collected by machines, investigators were not blinded during experiments.

Reporting for specific materials, systems and methods

Materials & experimental systems

n/a	Involved in the study	
x		Unique biological materials
	x	Antibodies
	x	Eukaryotic cell lines
x		Palaeontology
	x	Animals and other organisms
x		Human research participants

Methods

n/a	Involved in the study	
x		ChIP-seq
	x	Flow cytometry
x		MRI-based neuroimaging

Antibodies

Antibodies used	CD47 expression on tumor cells was determined by direct staining with AF647-conjugated rat anti-mouse CD47 antibody (clone MIAP301; rat IgG), or indirect staining with non-conjugated MIAP301 followed by goat anti-rat IgG antibody (clone Poly4054). For analysis of immune cell phenotypes, single cell suspensions were incubated with anti-mouse CD16/32 FcR blocking antibody (clone 93) and various combinations of the following fluorochrome-conjugated antibodies: anti-CD45 (clone 30-F11), CD11c (clone N418), CD11b (clone M1/70), SIRPα (CD172a; clone P84), CD3 (clone 17A2), CD4 (clone GK1.5), CD8 (clone 53-6.7), CD44 (clone IM7), CD62L (clone MEL-14), CD64 (clone X54-5/7.1), F4/80 (clone BM8), XCR1 (clone ZET), I-Ab (clone AF6-120.1), CD40 (clone 3/23) and CD86 (clone GL-1). Dead cells were identified by staining with propidium iodide or DAPI.
Validation	All the antibodies used in this study were commercially available, and have been validated by the vendors. Each of the antibodies were serially diluted and stain target cells to determine the titration.

Eukaryotic cell lines

Policy information about cell lines

Cell line source(s)	All cell lines were purchased from ATCC.
Authentication	Cell lines were authenticated by ATCC.
Mycoplasma contamination	All cell lines were tested negative for Mouse Essential Panel with PVM, provided by Charles River laboratory.

Commonly misidentified lines (See ICLAC register)	No commonly misidentified cell lines were used in this study.

Animals and other organisms

Policy information about studies involving animals; ARRIVE guidelines recommended for reporting animal research

Laboratory animals	Relevant information regarding animals and animal experiments are described in the methods of manuscript.
Wild animals	No wild animals were used.
Field-collected samples	No field-collected samples were used.

Flow Cytometry

Plots

Confirm that:

- [x] The axis labels state the marker and fluorochrome used (e.g. CD4-FITC).
- [x] The axis scales are clearly visible. Include numbers along axes only for bottom left plot of group (a 'group' is an analysis of identical markers).
- [x] All plots are contour plots with outliers or pseudocolor plots.
- [x] A numerical value for number of cells or percentage (with statistics) is provided.

Methodology

Sample preparation	Details of sample preparation were described in the methods section.
Instrument	BD Fortessa flow cytometer (BD Biosciences).
Software	Flow cytometry data was collected by BD FACSDiva and analyzed by FlowJo software v10.
Cell population abundance	The purity of sorted CD47KO B16F0 cells was >99%.
Gating strategy	The preliminary gating strategy was as follows: at first dead cells were excluded by gating on propidium iodide or DAPI negative population, then used FSC and SSC to exclude debris, and used FSC-A and FSC-H to exclude doublets. Different combinations of markers were used to define specific cell populations as described in the methods section.

- [x] Tick this box to confirm that a figure exemplifying the gating strategy is provided in the Supplementary Information.
